# iGHBP: Computational identification of growth hormone binding proteins from sequences using extremely randomised tree

**DOI:** 10.1016/j.csbj.2018.10.007

**Published:** 2018-10-24

**Authors:** Shaherin Basith, Balachandran Manavalan, Tae Hwan Shin, Gwang Lee

**Affiliations:** a Department of Physiology, Ajou University School of Medicine, Suwon, Republic of Korea; b Institute of Molecular Science and Technology, Ajou University, Suwon, Republic of Korea

**Keywords:** Extremely randomised tree, Growth hormone binding protein, Machine learning, Random forest, Support vector machine

## Abstract

A soluble carrier growth hormone binding protein (GHBP) that can selectively and non-covalently interact with growth hormone, thereby acting as a modulator or inhibitor of growth hormone signalling. Accurate identification of the GHBP from a given protein sequence also provides important clues for understanding cell growth and cellular mechanisms. In the postgenomic era, there has been an abundance of protein sequence data garnered, hence it is crucial to develop an automated computational method which enables fast and accurate identification of putative GHBPs within a vast number of candidate proteins. In this study, we describe a novel machine-learning-based predictor called iGHBP for the identification of GHBP. In order to predict GHBP from a given protein sequence, we trained an extremely randomised tree with an optimal feature set that was obtained from a combination of dipeptide composition and amino acid index values by applying a two-step feature selection protocol. During cross-validation analysis, iGHBP achieved an accuracy of 84.9%, which was ~7% higher than the control extremely randomised tree predictor trained with all features, thus demonstrating the effectiveness of our feature selection protocol. Furthermore, when objectively evaluated on an independent data set, our proposed iGHBP method displayed superior performance compared to the existing method. Additionally, a user-friendly web server that implements the proposed iGHBP has been established and is available at http://thegleelab.org/iGHBP.

## Highlights

• We developed an identification method for GHBPs using extremely randomized tree.
• iGHBP displayed superior performance compared to the existing method.
• We constructed a user-friendly web server that implements the proposed iGHBP method.

## 1 Introduction

Circulating growth hormones (GH) exist in a partially complexed form with binding proteins. The high affinity growth hormone binding protein (GHBP) is one such predominant GH binding protein that represents the extracellular ligand-binding domain of the GH receptor (GHR) [[1], [2], [3], [4]]. In humans, GHBP is generated by the proteolytic cleavage of the GHR at the cell surface using the tumor necrosis enzyme factor-α-converting enzyme (TACE), thereby releasing the extracellular domain of GHR (i.e., GHBP) [[5], [6], [7]]. By contrast, GHBP is produced in rodents by the alternative processing of the GHR transcript [8]. Binding GH to the GHR triggers the physiological functions of the hormone. Previous studies suggested that the biological effects of GHBP is dependent on the serum level of GH [5], as low levels of GH lead to a dwarf phenotype but increases the life longevity [1,9], while high levels lead to acromegaly, kidney damage, and diabetic eye. Therefore, the study of GHBP is gaining momentum from functional proteomics, leading to its clinical identification.

Traditionally, GHBPs were identified and characterised using biochemical experiments including immunoprecipitation, ligand immunofunctional assays, chromatography, and cross-linking assays [[10], [11], [12], [13]]. To identify GHBP from a protein sequence using these methods seems to be highly expensive, time-consuming, and overly complex to be utilised in a high-throughput manner. Thus, the development of sequence-based computational methods is needed to identify potential GHBP candidates. Recently, Tang et al. developed an Support vector machine (SVM)-based prediction model called HBPred [14], where the authors have used an optimal feature set obtained from dipeptide composition (DPC) using an incremental feature selection strategy. HBPred is the only publicly available method, which was developed using the same data set as our method. Although the existing method has a specific advantage in GHBP prediction, the accuracy and transferability of the prediction model still require improvement.

**Fig. 1:**
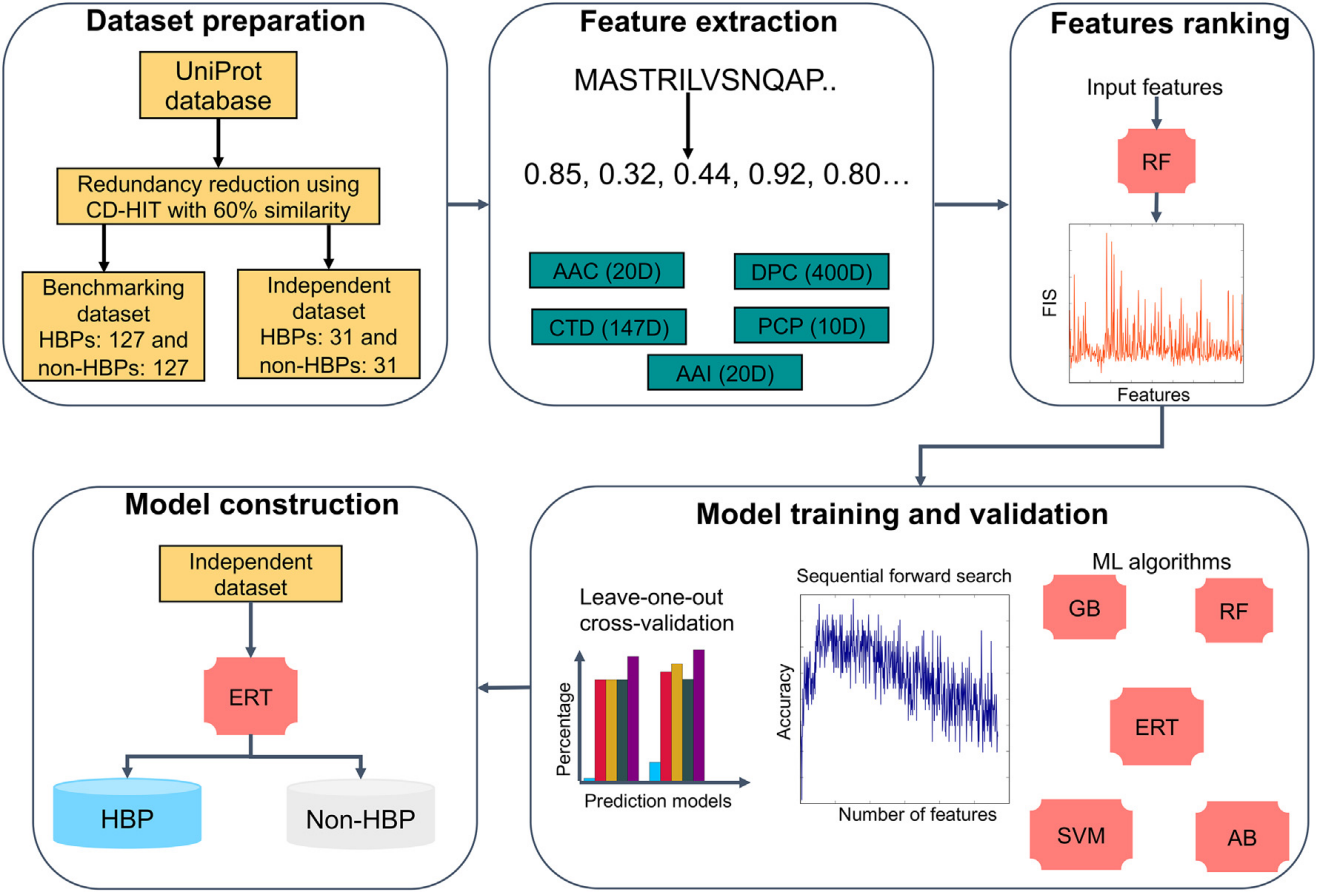
Overview of the proposed methodology for predicting GHBPs that involved the following steps. (i) data set construction; (ii) feature extraction; (iii) feature ranking; (iv) exploration of various machine learning algorithms and an appropriate selection based on the performance produced using sequential forward search; (v) construction of the final prediction model that separates the input into putative GHBPs and non-GHBPs.

In this study, we proposed a novel sequence-based predictor, called iGHBP, for the identification of GHBPs from given protein sequences (Fig. 1). Firstly, we collected GHBPs from UniProt and constructed nonredundant benchmarking and independent data sets. Secondly, we investigated five different machine learning (ML) algorithms [SVM, random forest (RF), extremely randomized tree (ERT), gradient boosting (GB), adaBoost (AB)], five compositions [amino acid composition (AAC), amino acid index (AAI), DPC, chain-transition-distribution (CTD), and physiochemical properties (PCP)], and 16 hybrid features (a linear combination of various compositions). In total, we generated 21 models for each ML method and selected the best model. Thirdly, we applied a two-step feature selection protocol on the above selected best model to improve the prediction performance. Finally, we evaluated these models against the state-of-the art method, HBPred, on the independent data set. Experimental results showed that the ERT-based prediction model produced consistent performance on both the benchmarking and independent data sets, hence, we named iGHBP as the superior model, demonstrated by outperforming the existing predictor as well as other predictors tested in this study. Therefore, it can be expected that iGHBP can be an effective tool for identifying GHBPs.

## 2 Methods

The iGHBP methodology development involved five major stages: Data set construction, feature extraction, feature ranking, model training and validation, and the construction of the final prediction model. Each of these major stages is described in the following section.

### 2.1 Data set construction

#### 2.1.1 Benchmarking data set

We utilised the data set constructed by Tang et al., [14] which was specifically used for the classification of GHBPs or non-GHBPs. The reason for considering this data set is as follows: (*i*) they have applied several filtering schemes to construct such a reliable data set; (*ii*) it is an nonredundant data set, and none of the sequences possesses pairwise sequence identity (>60%) with any other sequence; (*iii*) furthermore, it enables a fair comparison between our method and the existing method, which was developed using the same benchmarking data set. Thus, the benchmarking data set can be formulated as:

(1) $$S=S^{+}\cup S^{-}$$

where the subsets *S*^+^ and *S*^−^ respectively contain 123 GHBPs and 123 non-HBPs, and the symbol ∪ denotes a union, in set theory.

#### 2.1.2 Independent data set

To assess the performance of iGHBP with other related methods, we constructed an independent data set. Firstly, we considered 355 manually annotated and reviewed GHBP proteins from Universal Protein Resource (UniProt) using hormone-binding keywords in molecular function item of Gene Ontology. After this, we used CD-HIT [15], which is widely used to perform sequence clustering and to remove highly similar sequences, by setting a threshold of 0.6. The final data set contained 31 GHBPs and was supplemented with an equal number of non-GHBPs. Basically, these non-GHBPs are other functional proteins such as cancer lectins and phage virion proteins. Note that none of the protein sequences in the independent data set appeared in the benchmarking data set, ensuring a fair comparison of prediction model performance.

### 2.2 Feature representation of proteins

A protein sequence (P) can be represented as:

(2) $$P=R_{1}R_{2}R_{3}\ldots ..R_{N}$$

where *R*_1_, *R*_2_ and *R*_3_ respectively denote the 1^st^, 2^nd^, and 3^rd^ residues in the protein *P* and so forth. *N* denotes the protein length. It should be noted that the residue *R*_*i*_ is an element of the standard amino acid {A, C, D, E, F, G, H, I, K, L, M, N, P, Q, R, S, T, V, W, Y}. To develop a ML model, we formulated proteins with diverse-length as fixed-length feature vectors. We exploited five different compositions that cover different aspects of sequence information as described below:

#### 2.2.1 AAC

AAC is the percentage of standard amino acids; it has a fixed length of 20 features. AAC can be formulated as follows:

(3) $$\mathrm{AAC}\left(P\right)=\left(f_{1},f_{2},f_{3},\ldots \ldots ,f_{20}\right)$$

where $f_{i}=\frac{R_{i}}{N}\left(i=1,2,3,\ldots ,20\right)$ is the percentage of the composition with amino acid type *i*, *R*_*i*_ is the quantity of type *i* appearing in the protein, and *N* is the protein length.

#### 2.2.2 DPC

DPC is the rate of dipeptides normalised by all possible dipeptide combinations; it has a fixed length of 400 features. DPC can be formulated as follows:

(4) $$\mathrm{DPC}\left(P\right)=\left(f_{1},f_{2},f_{3},\ldots \ldots ,f_{400}\right)$$

where $f_{i}=\frac{R_{i}}{\sum_{i=1}^{400}R_{i}}\left(i=1,2,3,\ldots ,400\right)$ is the percentage of the composition with dipeptide type *i* and *R*_*i*_ is the quantity of type *i* appearing in the protein.

#### 2.2.3 CTD

CTD was introduced by Dubchak, et al. [16] for predicting protein-folding classes. A detailed description of computing CTD features was presented in our previous study [37]. Briefly, the twenty standard amino acids are classified into three different groups, namely: polar, neutral, and hydrophobic. Composition (C) consists of percentage composition values from these three groups for a target protein. Transition (T) consists of the percentage frequency of a polar followed by a neutral residue, or that of a neutral followed by a polar residue. This group may also contain a polar followed by a hydrophobic residue or a hydrophobic followed by a polar residue. Distribution (D) consists of five values for each of the three groups, and measures the percentage of a target sequence length within which 25, 50, 75, and 100% of the amino acids of a specific property are located. CTD generates 21 features for each PCP; hence, seven different PCPs (hydrophobicity, polarisability, normalised van der Waals volume, secondary structure, polarity, charge, and solvent accessibility) yield a total of 147 features.

#### 2.2.4 AAI

The AAIndex database contains a variety of physiochemical and biochemical properties of amino acids [17]. However, utilising all the information present in the AAIndex database as input features to the ML algorithm may affect the model’s performance, due to redundancy. To this end, Saha et al., [18] applied a fuzzy clustering method on the AAIndex database and classified it into eight clusters, where the central indices of each cluster were considered as high-quality amino acid indices. The accession numbers of the eight amino acid indices in the AAIndex database are BLAM930101, BIOV880101, MAXF760101, TSAJ990101, NAKH920108, CEDJ970104, LIFS790101, and MIYS990104. These high-quality indices encode the target protein sequences as 160-dimensional vectors. However, the average of these eight high-quality amino acid indices (a 20-dimensional vector) was used as an additional input feature to save the computational time.

#### 2.2.5 PCP

PCP computed from the target protein sequence includes: (*i*) hydrophobic residues (i.e., F, I, W, L, V, M, Y, C, A); (*ii*) hydrophilic residues (i.e., S, Q, T, R, K, N, D, E); (*iii*) neutral residues (i.e., H,G, P); (*iv*) positively charged residues (i.e., K, H, R); (*v*) negatively charged residues (i.e., D, E); (*vi*) *n* (sequence length); (*vii*) fraction of turn-forming residues (i.e., [N+G+P+S]/n); (*viii*) absolute charge per residue ($\left|\frac{R+K-D-E}{n}-0.03\right|$); (*ix*) molecular weight; and (*x*) aliphatic index (i.e., [A+2.9V+3.9I+3.9L]/n).

Briefly, we extracted five feature encoding schemes based on composition and physicochemical properties, which includes AAC, DPC, CTD, AAI, and PCP respectively generates 20-, 400-, 147-, 20-, and 10-dimensional vectors.

### 2.3 Machine learning algorithms

In this study, we explored five different ML algorithms, including RF, ERT, SVM, GB, and AB for binary classification (GHBP or non-GHBP). All these ML algorithms were implemented using the Scikit-Learn package (v0.18) [19]. A brief description of these methods and how they were used given in the following sections:

#### 2.3.1 Random forest

RF is one of the most successful ML methods, and utilises hundreds or thousands of independent decision trees to perform classification and regression [20]. RF combines the concepts of bagging and random feature selection. For a given training data set (*D*), generate a new training data set (*D*_*i*_) by uniformly drawing N bootstrapped samples from *D*. Grow a tree using *D*_*i*_ and repeat the following steps at each node of the tree until its fully grown: (*i*) select *mtry* random features from the original features and select the best variable by optimising the impurity criteria, and (*ii*) split the node into two child nodes. The tree grows until the amount of data in the node is below the given threshold (*nsplit*). Repeat the above-mentioned steps to build a large quantity (*ntree*) of classification trees. To classify a test data, input features are passed through from the root to the end node of each tree based on predetermined splits. The majority of the class from the forest is considered as the final classification.

#### 2.3.2 Extremely randomised tree

Geurts et al. [21] proposed the ERT algorithm, which utilises hundreds or thousands of independent decision trees to perform classification and regression problems, and has been applied in a large number of biological problems [22,23]. ERT aims to further decrease the variance of the prediction model by including stronger randomisation techniques. The ERT algorithm is similar to RF, but with the following differences: (*i*) ERT does not apply a bagging procedure for the construction of each tree. Instead, it uses the whole input training set for the construction of each tree. (*ii*) ERT selects a node split very randomly (both a variable index and variable splitting values are chosen randomly), whereas RF finds the best split (optimised by a variable index and a variable splitting value) among a random subset of variables. Furthermore, Grid search was performed for optimising the regularisation parameters *ntree*, *mtry*, and *nsplit*. The search space for *ntree*, *mtry*, and *nsplit* are:

(5) 40≤ntree≤1000with step ∆ntree=201≤mtry≤15with step ∆mtry=11≤nsplit≤10with step ∆nsplit=1

#### 2.3.3 Support vector machine

SVM is a well-known supervised ML algorithm [24], which has been widely used in various biological problems [25,26]. It maps the original feature vectors into a higher Hilbert space using different kernel functions and then searches an optimal hyperplane in Hilbert space. In this study, radial basis kernel function was utilized to construct a SVM model. Grid search was performed for optimizing regularisation parameters *C* and the kernel width parameter γ with the search space as mentioned in [27].

#### 2.3.4 Adaptive boosting

Fruend [28] proposed AB algorithm that combines a several weak classifiers to build a strong classifier. In this study, we treated decision tree as a base classifier with the default parameters as implemented in Scikit package. However, the number of estimators at which boosting terminated is optimized in the range of 50–500 with an interval of 50.

#### 2.3.5 Gradient boosting

Friedman proposed the GB algorithm [29], which is a forward learning ensemble method that produces a final strong prediction model based on the ensemble of weak models (decision trees), which has been widely used in bioinformatics and computational biology [27,30]. In GB, the two most influential parameters are *ntree*, and *nsplit*, we optimized with the search space as mentioned in [27].

In addition to the above ML algorithms, we note that there are other ML algorithms such as deep belief network, recurrent neural network, deep learning, and two-layer neural network have been successfully applied in various biological problems [[31], [32], [33], [34], [35], [36]]. However, these methods will be considered in our future studies.

### 2.4 Cross-validation

Generally, three cross-validation methods, namely an independent data set test, a sub-sampling (or *k*-fold cross-validation) test, and a leave-one-out cross-validation (LOOCV) test, are often used to evaluate the anticipated success rate of a predictor. Among the three methods, however, the LOOCV test is deemed the least arbitrary and most objective as demonstrated by Eqs. 28-32 of [37], and hence it has been widely recognised and increasingly adopted by investigators to examine the quality of various predictors [[38], [39], [40], [41], [42], [43], [44], [45], [46], [47], [48]]. Accordingly, the LOOCV test was also used to examine the performance of the model proposed in the current study. In the LOOCV test, each sequence in the training data set is in turn singled out as an independent test sample and all the rule-parameters are calculated without including the one being identified.

### 2.5 Performance evaluation

To evaluate the performance of the constructed models, we used four measurements that were commonly used in binary classification tasks, including sensitivity, specificity, accuracy, and Matthews correlation coefficient (MCC). They are calculated as follows:

$$\mathrm{Sensitivity}=\frac{\mathrm{TP}}{\mathrm{TP}+\mathrm{FN}}$$

$$\mathrm{Specificity}=\frac{\mathrm{TN}}{\mathrm{TN}+\mathrm{FP}}$$

$$\mathrm{Accuracy}=\frac{\mathrm{TP}+\mathrm{TN}}{\mathrm{TP}+\mathrm{TN}+\mathrm{FP}+\mathrm{FN}}$$

(6) $$\mathrm{MCC}=\frac{\mathrm{TP}\times \mathrm{TN}-\mathrm{FP}\times \mathrm{FN}}{\surd \left(\mathrm{TP}+\mathrm{FP}\right)\left(\mathrm{TP}+\mathrm{FN}\right)\left(\mathrm{TN}+\mathrm{FP}\right)\left(\mathrm{TN}+\mathrm{FN}\right)}$$

where TP is the number of true positives (i.e., GHBPs classified correctly as GHBPs) and TN is the number of true negatives (i.e., non-GHBPs classified correctly as non-GHBPs). FP is the number of false positives (i.e., GHBPs classified incorrectly as non-GHBPs) and FN is the number of false negatives (i.e., non-GHBPs classified incorrectly as GHBPs).

Additionally, the receiver operating characteristic (ROC) curve, which is a plot of the true positive rate against the false positive rate under different classification thresholds, is depicted to visually measure the comprehensive performance of different classifiers.

### 2.6 Feature selection

To improve the feature representation capability and identify the subset of optimal features that contribute for correctly classifying GHBPs and non-GHBPs, we employed a novel two-step feature selection strategy. Notably, the two-step feature selection protocol employed here is similar to the one used in our recent studies [26,[49], [50], [51]], where the features were ranked according to feature importance scores (FISs) using the RF algorithm in the first step, and feature subsets were selected manually based on the FISs in the second step. In this study, the first step is identical to our previous protocol. However, in the second step, a sequential forward search (SFS) was employed to select the optimal feature subset, rather than using manual feature subset selection.

In the first step, we inputted a given set of features for the RF algorithm and carried out a 10-fold cross-validation (CV). For each round of CV, we built 1000 trees using a *mtry* range from 1–50. The average FISs from all the trees were used to rank the features.

(7) $$D={\left[F1,F2,F3,\ldots \ldots ,\mathrm{FN}\right]}^{T}$$

where F1 is the first feature with the maximum FIS; F2 is the second feature with the second maximum FIS; F3 is the third feature with the third maximum FIS and so on; N and T are the total number of features and the transpose operator, respectively.

In the second step, we utilised SFS to identify and select the optimal features from a ranked feature set based on the following steps. (*i*) The first feature subset only contained the first feature in the ranked set D. The second feature subset contains the first and the second feature in D, and so on. Finally, we obtained N feature subsets. (*ii*) All the N feature subsets were inputted to ERT to develop their corresponding prediction model using a LOOCV test. Finally, the best performance produced by the feature subset was considered as the optimal feature set.

## 3 Results and discussion

### 3.1 Performance comparison of various models using different feature encodings

**Fig. 2:**
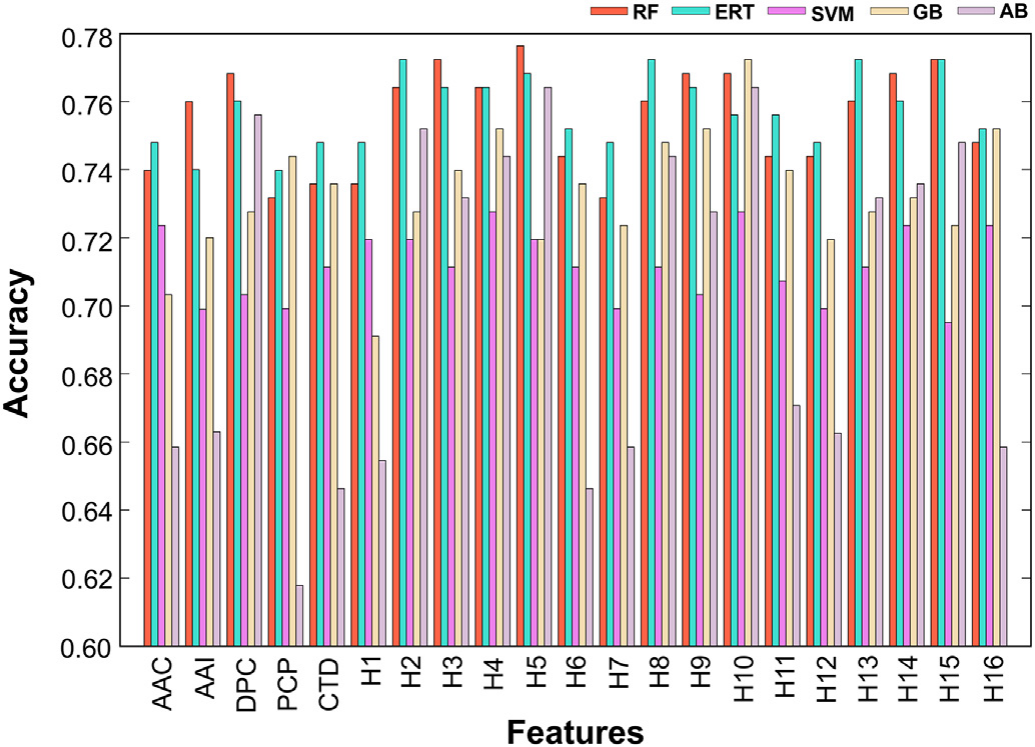
Performance of different ML-based models using the benchmarking data set. AAC: amino acid composition; DPC: dipeptide composition; CTD: chain-transition-distribution; AAI: amino acid index; PCP: physicochemical properties; H1: AAC + AAI; H2: AAC + DPC + AAI; H3: AAC + DPC + AAI + CTD; H4: AAC + DPC + AAI + CTD + PCP; H5: AAC + DPC; H6: AAC + CTD; H7: AAC + PCP; H8: AAI + DPC; H9: AAI + DPC + CTD; H10: AAI + DPC + CTD + PCP; H11: AAI + CTD; H12: AAI + PCP; H13: DPC + CTD; H14: DPC + CTD + PCP; H15: DPC + PCP; and H16: CTD + DPC.

In this study, we considered 21 feature encodings that include individual composition-based features and hybrid features (a linear combination of different individual compositions), which were inputted to five different ML algorithms, developing their corresponding models using a LOOCV procedure. In total, 105 prediction models were developed and the performance of each model in terms of accuracy with respect to the different feature encodings and ML algorithms is shown in Fig. 2. Among these methods, ERT and RF perform consistently better than other three algorithms (SVM, GB, and AB). Here, the model that achieved the highest accuracy was regarded as the best model. Accordingly, five models were selected from each ML method. Surprisingly, these five ML models produced their best performances using hybrid features (ERT: H8 (DPC+AAI); RF and AB: H5 (DPC+AAC); SVM: H4 (AAC+DPC+AAI+CTD+PCP); and GB: H10 (DPC+AAI+CTD+PCP)), indicating that various aspects of sequence information may be needed for a better prediction. Table 1 shows the performance comparison of five different ML methods, where the methods are ranked according to MCC and it can be considered as one of the best measures in binary classification [22,52]. Among these methods, RF, ERT, and GB produced a similar performance with an MCC and accuracy of 0.546 and 0.772, respectively, which is slightly better than AB and significantly better than SVM. Therefore, we selected only four ML-based models (RF, ERT, AB, and GB) and applied feature selection protocol on these models.

**Table 1:** The performance of the best model for each ML method obtained from different feature encodings.

Methods	Features	MCC	Accuracy	Sensitivity	Specificity	AUC
ERT	H8 (420)	0.546	0.772	0.740	0.805	0.813
RF	H5 (420)	0.546	0.776	0.829	0.724	0.805
GB	H10 (577)	0.545	0.772	0.789	0.756	0.806
AB	H5 (420)	0.531	0.764	0.715	0.813	0.767
SVM	H4 (597)	0.457	0.728	0.772	0.683	0.746

The first column represents the method name developed in this study. The second column represents the hybrid model and its corresponding number of features. The third, fourth, fifith, sixth, and seventh columns, respectively, represent the MCC, accuracy, sensitivity, specificity, and AUC. RF: random forest; ERT: extra tree classifier; SVM: support vector machine; GB: gradient boosting; and AB: adaBoost.

### 3.2 Construction of iGHBP

**Fig. 3:**
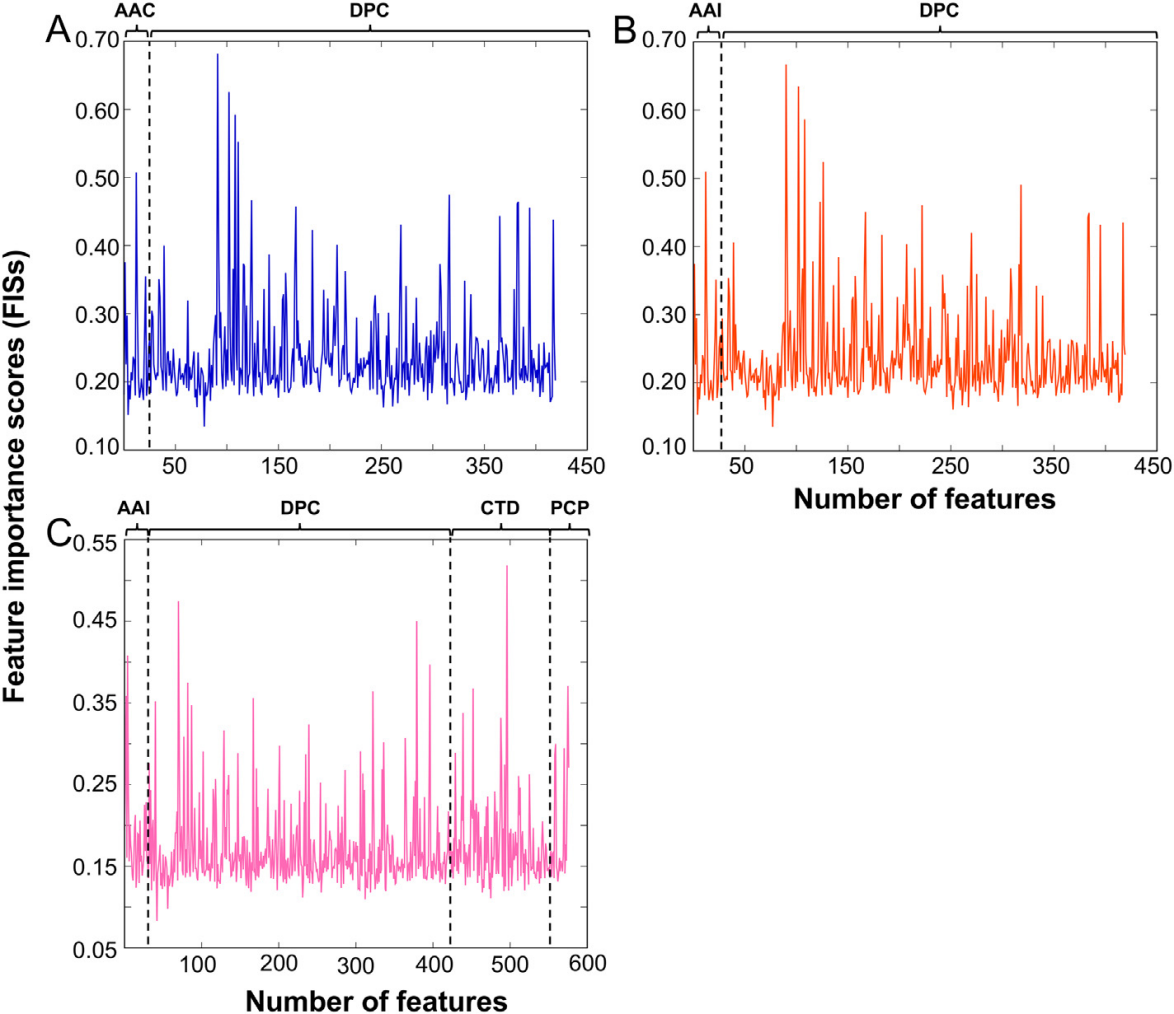
Feature importance score computed for the hybrid features H5 (A), H8 (B) and H10 (C) using the RF algorithm.

To identify the most informative features that improves a prediction performance, a feature selection protocol was employed to remove noisy and redundant features [[53], [54], [55], [56]]. In an effort to construct the optimal or best predictive model, we applied a two-step feature selection protocol to identify an optimal feature set from the hybrid features that improves the prediction performance. In the first step, we applied the RF algorithm to rank the features, according to FIS, with hybrid features H5 (Fig. 3A), H8 (Fig. 3B) and H10 (Fig. 3C).

**Fig. 4:**
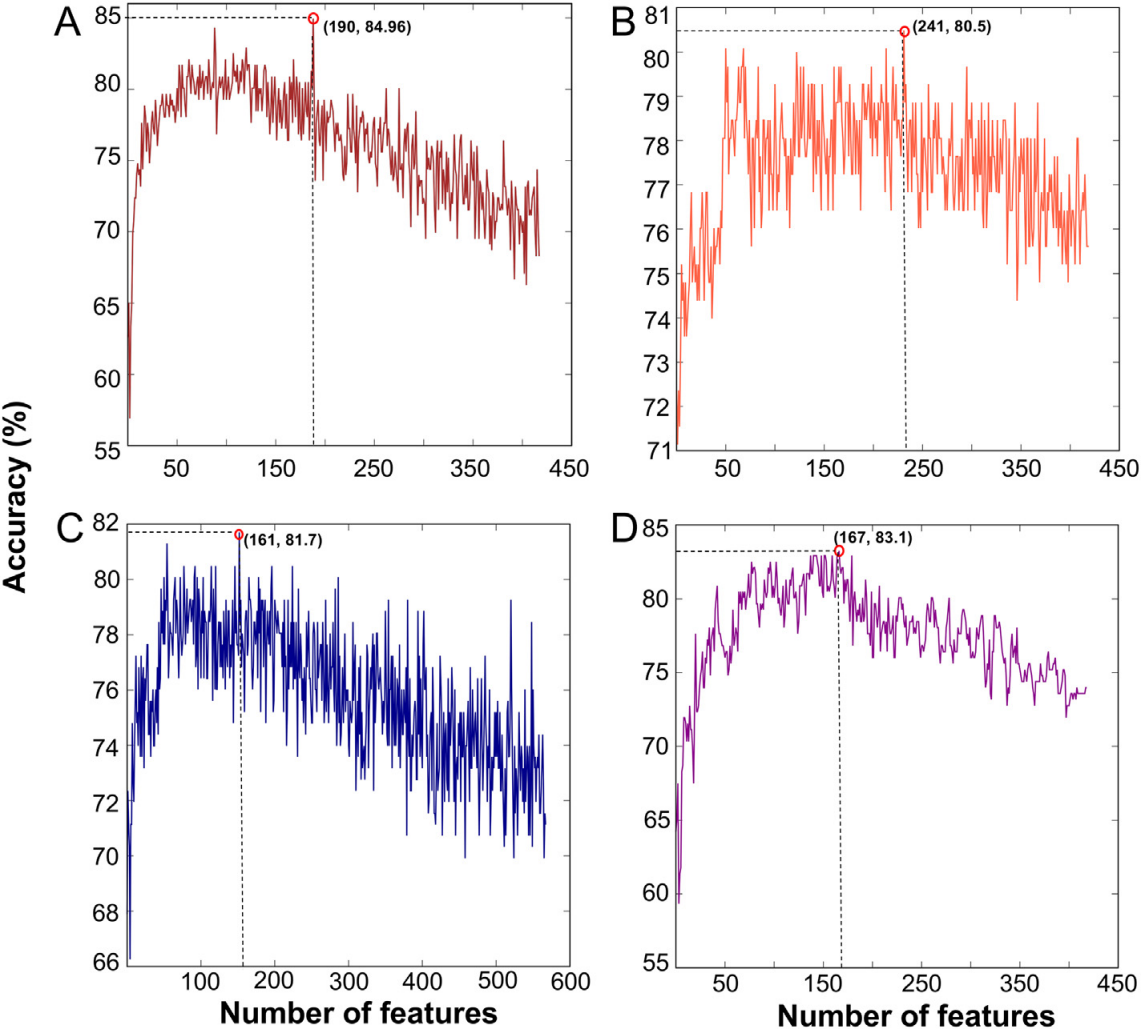
SFS curve for discriminating GHBPs and non-GHBPs. (A) -. The maximum accuracy (i.e., SFS peak) obtained in leave-one-out cross-validation is shown in the red circle.

SFS approach was used in the second step to select the optimal feature set from the ranked feature list. Fig. 3A shows the feature importance scores of 420-dimensional vectors. These features were ranked according to FIS and generated 420 feature sets (see methods). Each feature set was inputted to the ERT algorithm, and their corresponding models were developed using an LOOCV test. We plotted the SFS curve in Fig. 4A by using accuracy as Y-axis and feature number as X-axis. The maximum accuracy of 84.96% was observed with an optimal feature set of 190 features, while the other metrics such as MCC, sensitivity, specificity, and AUC are 0.701, 88.62, 81.30, and 0.896, respectively. Surprisingly, the obtained performance is identical to HBPred, where both methods use identical cross-validation methods and benchmarking data sets, however the number of features and the choice of ML algorithms are different. We also dramatically reduced the considered features from 420 to 190, indicating that our proposed feature selection technique could pick out the optimal dipeptides and AAI so as to further improve the prediction quality.

The above procedure was followed for other three methods (RF, GB, and AB). The best performance in terms of accuracy for RF, GB, and AB peaked at 80.5% (Fig. 4B), 81.7% (Fig. 4C), and 83.1% (Fig. 4D), respectively, with corresponding X-axis of 241, 161, and 167. These results show that a two-step feature selection protocol significantly improves the performances of the respective models. Next, we compared the performances of four different ML-based methods. To be specific, the accuracy of the ERT-based prediction model is ~1.9–4.4 higher than the other three methods, indicating the superiority of the ERT-based method in GHBP prediction. Hence, we named ERT-based prediction model as iGHBP.

### 3.3 Performance comparison between the optimal model and the control

**Fig. 5:**
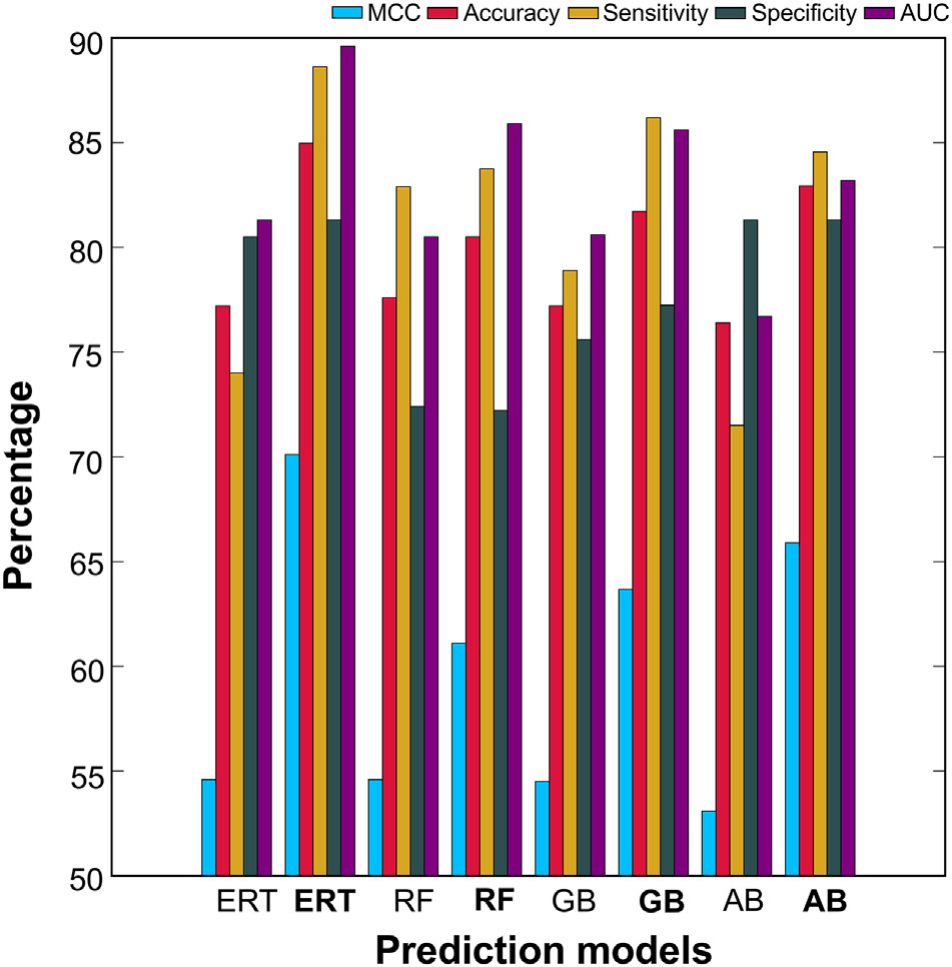
Performance comparison between the control (without feature selection) and optimal feature set-based models of four different ML algorithms. In the x-axis, normal and bold font respectively represent the control and the final model using the optimal feature set.

To show the efficiency of our feature selection protocol, we compared the performance of the optimal model and the control without feature selection or using all features. Fig. 5 shows that our two-step feature selection protocol significantly improved the prediction performances of all four ML-based methods. Specifically, ERT, RF, GB and AB, whose accuracy values were respectively 7.7%, 2.9%, 4.5%, and 6.6% higher than the control, indicating an effectiveness of feature selection protocol. A similar protocol has been used in previous studies and has shown that the corresponding optimal models improved in performance [53,54,56,57].

### 3.4 Analysis of feature selection

**Fig. 6:**
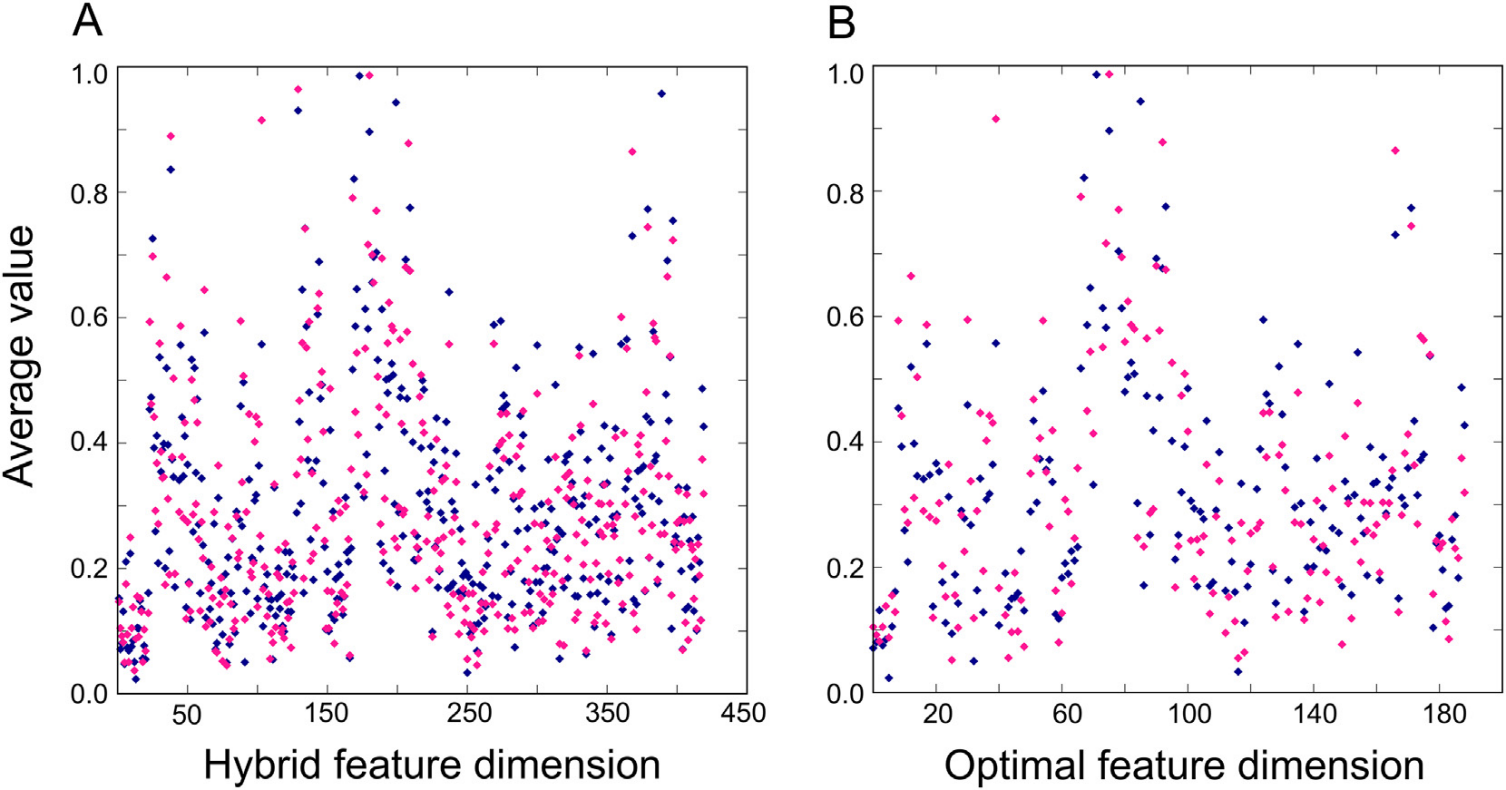
Distribution of the GHBPs and non-GHBPs in the benchmarking data set using our hybrid features (A) and the optimal feature set (B).

Although feature selection protocol significantly improved the performances of the respective ML-based methods, we specifically investigated the effectiveness of our feature selection protocol on ERT-based method (iGHBP). Here, we computed each feature average of GHBPs and non-GHBPs separately and compared their distribution for the hybrid features (Fig. 6A) and the optimal features (Fig. 6B). Results show that GHBPs and non-GHBPs were distributed more differentially in the feature space using optimal feature set when compared to the hybrid features, demonstrating why our feature descriptor led to the most informative prediction of GHBPs.

### 3.5 Performance assessment for GHBP prediction based on the independent data set

Generally, it is essential to evaluate the proposed model using an independent data set to check whether the prediction model has generalisation capability or robustness [26]. In order to check the robustness of iGHBP, we further compared against three other ML methods developed in this study and against the state-of-the-art predictor (HBPred) on the independent data set. To make a fair comparison, we ensure lower sequence identities between the benchmarking and independent data sets, as it would otherwise lead to an overestimation of performance if the sequences in the independent data set had higher identities that those in the benchmarking data set. The results are summarised in Table 2, where the methods are ranked according to MCC. It can be observed that the proposed predictor iGHBP achieved the best performance with the following metrics with MCC, accuracy, specificity, and AUC, values of 0.646, 82.3%, 83.9, and 0.813, respectively. Specifically, the MCC and accuracy of iGHBP were 17.4–45% and 9.7–22.6% higher when compared to the other methods, thus demonstrating the superiority of iGHBP. Furthermore, we computed a pairwise comparison of AUCs between iGHBP and HBPred using two-tailed*t* test [58] and obtained the *P*-value of 0.009, demonstrating iGHBP significantly outperformed the HBPpred.

**Table 2:** Performances of various methods on the independent data set.

Methods	Features	MCC	Accuracy	Sensitivity	Specificity	AUC
ERT	190	0.646	0.823	0.807	0.839	0.813
RF	241	0.472	0.726	0.871	0.581	0.777
GB	161	0.331	0.661	0.774	0.548	0.700
AB	167	0.324	0.661	0.613	0.710	0.675
HBPred	73	0.196	0.597	0.677	0.516	0.600

The first column represents the method name as used in this study. The second column represents the number of features present in the optimal feature set. The third, fourth, fifth, sixth and seventh columns, respectively, represent the MCC, accuracy, sensitivity, specificity, and AUC.

**Fig. 7:**
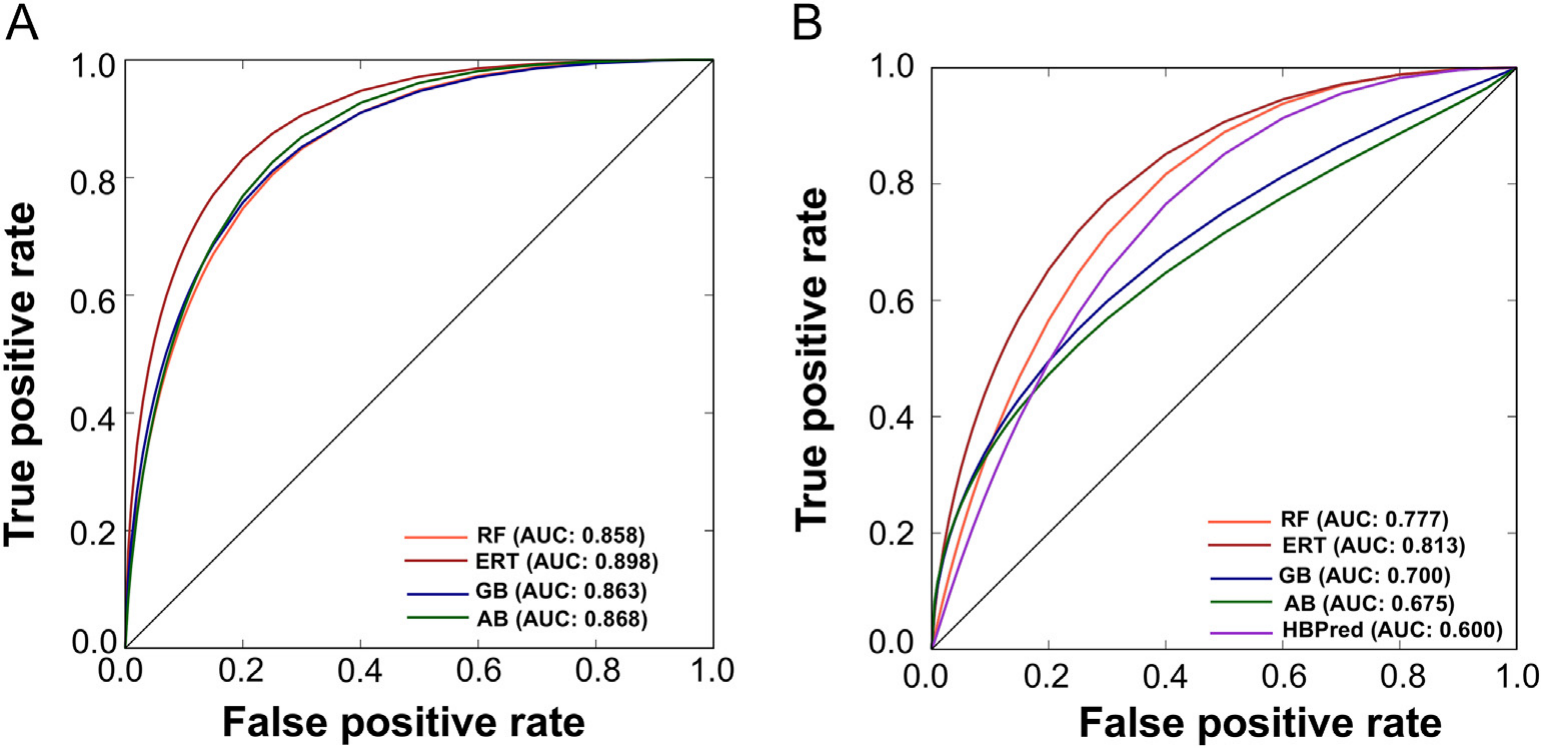
Receiver operating characteristic curves of the various prediction models. (A) Leave-one-out cross-validation on the benchmarking data set and (B) independent data set. Higher AUC value indicates better performance of a particular method.

It is worth mentioning that both iGHBP and HBPred produced identical performance with the benchmarking data set, although there was variation in the input feature dimension and ML algorithm. However, only iGHBP produced a similar and consistent performance in both the benchmarking and independent data sets (Fig. 7, A and B), indicating that the current predictor is more stable and reliable. Notably, the optimal feature set contains 190 features, which is ~ 3-fold higher than the features used in the previous study. It is understandable that a larger and optimal feature set plays an important role in capturing the key components between the actual GHBPs and non-GHBPs and improve the performance. This is remarkable progress in biological research because a more reliable tool for the identification of biological macromolecules can vastly reduce the experimental cost. Hence, the iGHBP can be expected to be a tool with a high availability for the identification of GHBPs.

### 3.6 Web server implementation

As pointed out in [37] and shown in many follow-up publications [25,49,[59], [60], [61], [62], [63], [64], [65], [66], [67], [68], [69], [70], [71], [72]], user-friendly and publicly accessible web servers are the future of direction for developing more useful predictors. To this end, an online prediction server for iGHBP was developed, and it is available at www.thegleelab.org/iGHBP. All data sets utilized in the current study can be downloaded from our web server. Below, we give researchers a step-by-step guideline on how to use the webserver to get their desired results. In the first step, users need to submit the query sequences into the input box. Note that the input sequences should be in FASTA format. Examples of FASTA-formatted sequences can be seen by clicking on the button FASTA format above the input box. Finally, clicking on the button Submit, you will get the predicted results on the screen of your computer.

## 4 Conclusions

The biological significance of GHBPs has motivated the development of computational tools that facilitate accurate prediction. In this work, we developed a novel GHBP predictor called iGHBP. Here, we systematically assessed the use and performance of various composition-based features and their combinations along with various ML approaches in GHBP prediction. Our main findings are as follows: (*i*) Among five classifiers, ERT performed the best according to our performance measures (MCC, accuracy, and AUC), based on LOOCV. (*ii*) Of those five different compositions, an optimal feature set using a combination of DPC and AAI achieved the highest performance, emphasising the arrangement of particular local ordering dipeptides and biochemical properties. (*iii*) Experiment results from independent tests show that the proposed predictor iGHBP is more promising and effective for the GHBPs identification. As an application of this method, we have also made available an iGHBP webserver for the wider research community to use. It is expected that iGBHP will be a useful tool for discovering hypothetical GHBPs in a high-throughput and cost-effective manner, facilitating characterisation of their functional mechanisms. Furthermore, our proposed methods, along with the increasing availability of experimentally verified data and novel features, will greatly improve the prediction of GHBP.

## Conflict of interest

The authors declare that there is no conflict of interest.

## Authors Contributions

BM and GL conceived and designed the experiments. SB and BM performed the experiments. BM, SB, and TS analyzed the data. GL contributed reagents/materials/software tools. BM, SB, and GL wrote the manuscript.
